# Patients’ Behavior Regarding Dietary or Herbal Supplements before and during COVID-19 in Saudi Arabia

**DOI:** 10.3390/ijerph18105086

**Published:** 2021-05-11

**Authors:** Leen A. Aldwihi, Shahd I. Khan, Faisal F. Alamri, Yazed AlRuthia, Faleh Alqahtani, Omer I. Fantoukh, Ahmed Assiri, Omar A. Almohammed

**Affiliations:** 1 Department of Clinical Pharmacy, College of Pharmacy, King Saud University, Riyadh 11451, Saudi Arabia; 436202414@student.ksu.edu.sa (L.A.A.); 441204353@student.ksu.edu.sa (S.I.K.); yazeed@ksu.edu.sa (Y.A.); 2 Pharmaceutical Care Department, AlNoor Specialist Hospital, Ministry of Health, Makkah 24241, Saudi Arabia; 3 Basic Sciences Department, College of Science and Health Professions, King Saud bin Abdulaziz University for Health Sciences, Jeddah 22384, Saudi Arabia; alamrif@ksau-hs.edu.sa; 4 King Abdullah International Medical Research Center, Jeddah 22384, Saudi Arabia; 5 Pharmacoeconomics Research Unit, College of Pharmacy, King Saud University, Riyadh 11451, Saudi Arabia; 6 Department of Pharmacology and Toxicology, College of Pharmacy, King Saud University, Riyadh 11451, Saudi Arabia; afaleh@ksu.edu.sa; 7 Department of Pharmacognosy, College of Pharmacy, King Saud University, Riyadh 11451, Saudi Arabia; ofantoukh@ksu.edu.sa; 8 General Directorate of Clinical Excellence, Ministry of Health, Riyadh 11176, Saudi Arabia; aasssiri@moh.gov.sa

**Keywords:** COVID-19, dietary, herbal, supplements, Saudi Arabia, behavior, hospitalization

## Abstract

The use of traditional medicinal plants in Saudi Arabia stems mainly from consumers’ belief in prophetic medicine. This study was conducted to explore changes in patients’ use of dietary or herbal supplements among individuals infected with COVID-19 before and during infection and the association between herbal or dietary supplements and hospitalization. A cross-sectional, questionnaire-based study was conducted enrolling symptomatic patients who had recently recovered from COVID-19. Data were collected through phone interviews, and McNemar’s test was used to investigate changes to consumption of dietary or herbal supplements before and during infection. Multivariable logistic regression was used to investigate the association between supplements use during patients’ infection and hospitalization. A total of 738 patients were included in this study, of whom 32.1% required hospitalization. About 57% of participants were male with a mean age of 36.5 (±11.9) years. The use of lemon/orange, honey, ginger, vitamin C, and black seed among participants significantly increased during their infection. In contrast, patients using anise, peppermint, and coffee peel before their infection were more likely to stop using them during their infection. In addition, using lemon/orange (*p* < 0.0001), honey (*p* = 0.0002), ginger (*p* = 0.0053), vitamin C (*p* = 0.0006), black seed (*p* < 0.0001), peppermint (*p* = 0.0027), costus (*p* = 0.0095), and turmeric (*p* = 0.0012) was significantly higher among nonhospitalized patients than hospitalized ones. However, in the multivariable logistic regression, only use of vitamin C (OR = 0.51; 95% CI 0.33–0.79), peppermint (OR = 0.53; 95% CI 0.31–0.90), and lemon/orange (OR = 0.54; 95% CI 0.33–0.88) was associated with significantly lower odds of hospitalization. The study reveals that patients’ consumption of dietary or herbal supplements changed in response to their COVID-19 infection, with hospitalized patients having a lower likelihood of using these supplements. Because some supplements were associated with lower odds of hospitalization, these supplements or their bioactive components should be further investigated as feasible options for COVID-19 treatment.

## 1. Introduction

Coronavirus disease 2019 (COVID-19) is the third-largest coronavirus outbreak of the century, after the outbreaks of severe acute respiratory syndrome (SARS) and Middle East respiratory syndrome (MERS) [1]. This outbreak was caused by the novel coronavirus, or severe acute respiratory syndrome coronavirus-2 (SARS-CoV-2), whose high level of transmissibility from person to person resulted in an epidemic worldwide. On March 11, the World Health Organization (WHO) classified COVID-19 as a pandemic disease [2]. Some patients infected with SARS-CoV-2 develop no symptoms (asymptomatic cases), and some have mild to moderate gastrointestinal or respiratory symptoms, but others develop severe pneumonia or even die [3]. The duration from onset to clinical recovery is unspecific and unpredictable. COVID-19 symptoms can vary from days to weeks to, in severe cases, months [4]. Although COVID-19 poses a lower risk of death than MERS and SARS, it has resulted in more deaths than both combined [5] because of the large number of COVID-19 cases. This is mainly due to the higher transmissibility of SARS-CoV-2 than SARS and MERS and its ability to transform to newer variants that became even more transmissible than the preexisting variants, which has resulted in a larger number of cases and consequently deaths compared to SARS and MERS [6,7].

Identifying reliable predictors and risk factors for disease severity, mortality, and hospitalization is crucial to saving patients’ lives. Moreover, understanding and studying community-level changes in health-related behaviors and beliefs during this pandemic is necessary, especially regarding self-medication, dietary habits, and the use of dietary or herbal supplements [8]. This will help provide recommendations, such as following a certain diet or supplementing with a certain product that may help decrease the number of severe cases and hospitalizations, thereby reducing the burden on the healthcare system and improving outcomes.

COVID-19 triggers an inflammatory immune response. Release of inflammatory cytokines in cases of COVID-19 leads to a cytokine storm and immune dysregulation, producing acute respiratory distress syndrome and multiorgan failure [9,10]. Accordingly, adopting healthy eating habits and using dietary or herbal supplements may support immunity and protect against adverse outcomes [11,12,13]. The available literature presents clear evidence supporting using herbal medicines against SARS-CoV-2 for their antiviral, anti-inflammatory, antioxidant, immune-supportive, and preventive effects [14]. Dietary and herbal supplements regulate immune function and control both adaptive and innate immunity in a multidirectional manner [15].

Antiviral activity of dietary or herbal supplements has been reported toward multiple virus strains, such as human immunodeficiency virus, hepatitis B and C viruses, herpes simplex virus, influenza viruses, and previous coronaviruses, such as SARS and MERS [16]. These supplements have a long history of treating respiratory infections and have been approved as over-the-counter drugs or nutraceuticals. In general, because these supplements are perceived as having minimal toxicity and favorable safety profiles, they are highly advisable for long-term usage [14]. Citrus fruits, such as lemon and orange, also have a promising therapeutic effect against COVID-19. Hesperidin, a bioactive flavanone glycoside abundant in citrus peels, stands out for its high binding affinity to the main cellular receptors of SARS-CoV-2, outperforming drugs already recommended for clinical trials. Thus it is promising for the prophylaxis and treatment of COVID-19 [17]. Moreover, lemon is an immunity-boosting food and can help relieve symptoms of the common cold, mitigate respiratory symptoms, throat colds, and pneumonia [18,19]. Peppermint is another plant species that could be exploited to prevent viral infections [20]. Similarly, curcumin has beneficial clinical effects, including antiviral, antinociceptive, anti-inflammatory, antipyretic, and antifatigue properties that could be of help in managing the symptoms of COVID-19 [21].

Interestingly, natural products were exploited in the development of a wide range of therapeutic agents. Specifically, 75% of medications in infectious diseases are naturally inspired compounds [22]. Moreover, it has been estimated that 80% of the population worldwide relies primarily on traditional remedies when seeking therapy [23]. Myriad factors have contributed to the public interest in dietary or herbal supplement use, such as established traditional use, reasonable cost, availability, and accessibility. In addition, a study from Spain that assessed the eating habit changes of the Spanish adult population found an improvement in eating behaviors during the COVID-19 pandemic [24].

Since the beginning of the COVID-19 pandemic, the use and sales of dietary supplements have increased globally [25]. Moreover, the Chinese government has been advising doctors to consider the use of Traditional Chinese medicine for COVID-19 patients in addition to standard care [26]. A recent systematic review and meta-analysis summarized results from seven trials investigating the benefits of using Chinese herbal medicine in treating COVID-19 compared to standard care (conventional use of Western medicine or routine medical care). The results clearly showed that adding Chinese herbal medicine to standard care improved the signs and symptoms, decreased the level of the inflammatory marker, and facilitated the absorption of lung infection lesions of COVID-19 patients [27]. Moreover, a retrospective single-center cohort study was conducted, including 300 patients with COVID-19 in China. Patients were divided into three different cohorts based on the timing of use for traditional Chinese medicine from their hospitalization; within 7 days, 8–14 days, and greater than 15 days. The main conclusion from this study was that early intervention with traditional Chinese medicine could promote faster recovery [28].

In Saudi Arabia, the public still uses herbal medicines to treat various health conditions or support general health in both urban and rural areas. Studies have reported a high prevalence of using dietary or herbal supplements for therapeutic purposes among adults in Saudi Arabia, with the most reported reasons for the use of herbal medicines involving traditional beliefs in their efficacy and safety as well as their affordability compared with other pharmaceutical products [29,30]. However, few studies have examined patients’ use of dietary or herbal supplements during the COVID-19 pandemic. Accordingly, this study explored changes in the use of dietary or herbal supplements among individuals with COVID-19 before and during their infection, assessing the association between the use of herbal or dietary supplements and hospitalization. Hopefully, the study’s findings can help highlight the significance of using herbal or dietary supplements during this pandemic and shed light on potential natural candidates for treating COVID-19 to allow further phytochemical and biological assessment.

## 2. Materials and Methods

### 2.1. Study Design and Participants

A cross-sectional, questionnaire-based study was conducted that included adult patients (≥18 years) in Saudi Arabia who could understand Arabic and who had recently recovered from COVID-19. Asymptomatic patients were excluded from the study. Participants were recruited from the Saudi Ministry of Health database of patients with a COVID-19 diagnosis confirmed by a polymerase chain reaction (PCR) test for SARS-CoV-2. Patients were informed about the nature and purpose of the study, and their consent was received verbally over the phone. Ethical approval was received from the Ministry of Health, Kingdom of Saudi Arabia (IRB No: 20-11E/17-06-2020), with the need for written consent waived by the ethical committee.

### 2.2. Data Collection

A random and stratified sample of patients was selected from the pool of recovered patients in the MoH database. Potential participants were then contacted over the phone. After they agreed to participate, questions were asked verbally, and the responses were documented by interviewers in an electronic data collection sheet. Data collection took place between August and October 2020. Potential cases were selected from various regions in Saudi Arabia to produce a diversified sample representing different areas of Saudi Arabia. Data on participants’ demographics, symptoms, consumption of dietary or herbal supplements before and during COVID-19 infection, clinical outcomes relating to their COVID-19 infection, and lifestyle variables were collected during the phone interview with consenting participants.

### 2.3. Questionnaire

Some dietary or herbal supplements exert antiviral, anti-inflammatory, antioxidant, immune supportive, or regulatory effects [14,15], making them candidates for fighting COVID-19 infection. Many of these supplements are used by the public in Saudi Arabia for their therapeutic effects, based mainly on traditional beliefs in their efficacy and safety [29,30]. The questionnaire in this study was constructed in Arabic by five members of the research team to explore changes in dietary or herbal supplements among individuals with COVID-19 before and during their infection. It consisted of 45 items divided into three sections. The first section featured 5 items about participants’ sociodemographic characteristics. The second section featured 13 items on the presence of comorbid conditions and the clinical aspects of the participants’ infection with COVID-19, such as their symptoms, the symptoms’ duration, and the need for hospitalization during their course of treatment. The third section featured 27 items about lifestyle choices, including the consumption of dietary or herbal supplements before and during their COVID-19 infection. Dietary or herbal supplements with known therapeutic effects were included in the study (details about these supplements are provided in the discussion.) Data on participants’ consumption of these supplements were captured using closed-ended dichotomous questions (“Yes, I used it”/“No, I did not use it”). The face validity for the questionnaire was validated by six expert healthcare professionals. Content validity was determined by two subject matter experts: a dietitian and a pharmacognosist. The Cronbach’s alpha coefficients for the internal consistency of the items pertaining to using supplements before and during infection with COVID 19 were 0.75 and 0.76, respectively.

### 2.4. Statistical Analysis

Descriptive statistics were used to describe patients’ characteristics and use of dietary or herbal supplements. Continuous variables (age and body mass index (BMI)) were reported as mean with standard deviation, whereas categorical variables were reported as frequencies with percentages. Inferential statistics such as chi-squared tests were used to assess differences between hospitalized and nonhospitalized participants in demographical variables along with the association between the use of dietary or herbal supplements during COVID-19 infection and hospitalization. McNemar’s tests were used to assess the significance of the change in dietary or herbal supplements’ consumption before and during infection with COVID-19. Multivariable logistic regression analysis was then used to investigate the association between patients’ use of dietary or herbal supplements during their infection and their hospitalization while controlling for the effects of age, gender, body mass index (BMI), marital status, and the presence of any of the following comorbid conditions: diabetes, hypertension, dyslipidemia, pulmonary diseases (e.g., asthma, COPD), cardiovascular diseases, chronic kidney diseases, cancer, an acquired immunodeficiency syndrome (AIDS), and depression or anxiety. The statistical significance of the results was predetermined at α < 0.05. All statistical analyses were conducted using SAS software version 9.4 (SAS Institute Inc., Cary, NC, USA).

## 3. Results

A total of 738 participants were included, more than half of whom were male (56.6%). The mean age and BMI for participants were 36.5 (± 11.9) and 28.4 (± 7.1), respectively. Most participants were Saudis (75.7%) and married (67.8%). Furthermore, 32.1% of participants had a severe COVID-19 infection and required hospitalization. Hospitalized patients were significantly older than nonhospitalized patients (42.4 vs. 30.6; *p* < 0.0001). Non-Saudis were more likely to be hospitalized during their recovery (48.6% vs. 26.8%; *p* = 0.0012). Furthermore, widows and widowers were more likely to be hospitalized during their recovery from COVID-19 than were single, married, or divorced participants (*p* = 0.0015). Table 1 gives the demographic characteristics for hospitalized and nonhospitalized participants.

### 3.1. Changes in Patients’ Use of Dietary or Herbal Supplements

Some dietary or herbal supplements were likely to be consumed by participants even before their infection with COVID-19, which may indicate their belief in the protective and immune-boosting effects of these supplements: lemon (Citrus × limon)/orange (Citrus × sinensis; 49.9%), honey (43.4%), and vitamin C (48.8%). Consumption of other supplements was less likely among participants before their infection with COVID-19. About 80% of the participants did not use black seed (*Nigella sativa*) as part of their lifestyle before their infection with COVID-19, whereas 92.7% of the participants had not used chamomile (*Matricaria chamomilla*), and 63.8% did not report using ginger (*Zingiber officinale*). Table 2 shows normal consumption of dietary or herbal supplements among participants before and during infection with COVID-19.

The overall use of dietary or herbal supplements dramatically increased among participants after their infection with COVID-19. The supplements associated with the most significant increase in the proportion of patients using them were lemon/orange (30.2%; *p* < 0.0001), followed by honey (27.6%; *p* < 0.0001) and black seed (27.1%; *p* < 0.0001) and then ginger (21.4%; *p* < 0.0001) and vitamin C (19.6%; *p* < 0.0001). By contrast, there was a significant reduction in participants’ consumption of other supplements, such as peppermint (*Mentha × piperita*; −13.4%, *p* < 0.0001). Table 2 summarizes these results.

Patients who used peppermint, chamomile, coffee peel (*Coffea arabica*), and cumin (*Cuminum cyminum*) before their SARS-CoV-2 infection were highly likely to stop or avoid using these supplements after their infection. Conversely, patients, who were not using ginger lemon/orange, honey, black seed, or vitamin C were highly likely to start using these supplements after being infected with SARS-CoV-2. Table 3 illustrates the changes in patients’ behavior during infection with COVID-19 for previous consumers or nonconsumers of dietary or herbal supplements.

### 3.2. The Association between Use of Dietary or Herbal Supplements during Infection with COVID 19 and Subsequent Hospitalization

There was a significant negative correlation between using some dietary or herbal supplements during COVID-19 infection and the proportion of patients hospitalized for treatment. Participants, who used lemon/orange (*p* < 0.0001), vitamin C (*p* = 0.0006), black seed (*p* < 0.0001), honey (*p* = 0.0002), turmeric (*Curcuma longa*; *p* < 0.0012), peppermint (*p* = 0.0027), ginger (*p* < 0.0053), or costus (*Saussurea costus*; *p* < 0.0095) during their COVID-19 infection were less likely to be hospitalized during their COVID-19 recovery, as Table 4 illustrates.

After adjusting the previous results for the effects of age, gender, BMI, marital status, and the presence of any of the comorbid conditions diabetes, hypertension, dyslipidemia, pulmonary diseases (e.g., asthma, COPD), cardiovascular diseases, chronic kidney diseases, acquired immunodeficiency syndrome (AIDS), cancer, and depression or anxiety in the multivariable logistic regression, only three supplements remained significant: vitamin C (odds ratio (OR) = 0.51; 95% CI: 0.33–0.79), peppermint (OR = 0.53; 95% CI: 0.31–0.90), and lemon/orange (OR = 0.54; 95% CI: 0.33–0.88), as Table 4 illustrates. Besides that, after the adjustment of the results for all previously mentioned variables using vitamin D was associated with higher odds of hospitalization (OR = 1.89; 95% CI: 1.20–2.98), but this became significant after adjusting for other factors, which could be the actual reason for hospitalization.

## 4. Discussion

Few studies have reported on using dietary or herbal supplements during infection with COVID-19 in Saudi Arabia. This study examined changes in the use of dietary or herbal supplements among individuals who contracted COVID-19, both before and during their infection. In addition, it assessed the association between using herbal and/or dietary supplements and hospitalization. The findings of this study may play a pivotal role in educating healthcare providers about the consumption of natural supplements when counseling their patients.

### 4.1. Citrus Fruits

Citrus fruits, such as lemon (*C. limon*) and orange (*C. sinensis*), are well-known for medicinal, cosmetic, and nutritional properties. The high content of phenolic compounds in citrus fruits, primarily flavonoids (e.g., diosmin, hesperidin, limocitrin) and phenolic acids (e.g., ferulic, synapic, *p*-hydroxybenzoic acids), determine their biological actions [31]. Essential oils of citrus fruits are used in traditional medicine to suppress coughs when mixed with sugar. The juice is traditionally used to treat scurvy, sore throat, fever, rheumatism, high blood pressure, and chest pain and is high in vitamin C, which supports the body when fighting infections [32]. Citrus fruits are an excellent vitamin C source, an immunomodulator, antifibrotic, and antioxidant. They have a potential therapeutic effect against many respiratory conditions, such as pulmonary fibrosis, pneumonia, and ARDS, as well as other conditions, including sepsis and acute lung, kidney, and liver injury [33]. In the present study, it was noticed that similar proportions of participants who consumed vitamin C or lemon/orange were hospitalized, with users of either having a lower odds of hospitalization than nonusers (lemon/orange: OR = 0.54; 95% CI: 0.33–0.88; vitamin C: OR = 0.51; 95% CI: 0.33–0.79).

### 4.2. Vitamin C

Vitamin C, or ascorbic acid, has significant antioxidant and immunomodulatory properties: it accumulates in neutrophils, increasing phagocytosis, natural killing cell activity, and lymphocyte proliferation [34]. A cross-sectional study was conducted in Saudi Arabia using an online survey to explore knowledge about COVID-19 preventive methods and opinions about the consumption of herbal supplements to prevent COVID-19 infection. A total of 5258 individuals participated in this study, about 22% of whom reported that they had used herbal products or nutritional supplements during the pandemic to protect themselves from the disease. In addition, vitamin C was the most commonly used supplement to enhance immunity and decrease the chances of contracting COVID-19 [35]. In the current study, 68% of patients who had COVID-19 used vitamin C during their infection. Moreover, participants who used vitamin C during their infection had a lower odds of hospitalization during their recovery from COVID-19 (OR = 0.51; 95% CI: 0.33–0.79).

### 4.3. Black Seed

Black seed (*N. sativa*) has been used in prophetic and traditional Arabic herbal medicine, especially in the Middle East, for more than 2000 years to treat various diseases, including skin diseases, asthma, cough, bronchitis, headache, fever, and influenza [36]. The composition of black seed includes fixed oils, proteins, alkaloids, saponins, and essential oils. However, most pharmacological activities of black seed are attributed to the presence of thymoquinone [37]. The impact of black seed oil on patients who had hepatitis C virus (HCV) infection and who were not eligible for interferon therapy was assessed in a study conducted in Egypt. A dose of 450 mg of black seed oil was administered to these patients as soft gelatin capsules three times daily for three months and resulted in a significant reduction in the HCV viral load [38]. In the current study, using black seed during infection with COVID-19 has significantly increased among the public by 27%. Although the results indicated a slight reduction in the need for hospitalization among the users of the black seed, this association was not significant in the multivariable logistic regression. A prospective, randomized, exploratory phase II clinical trial is currently being conducted by researchers from King Abdul-Aziz University in Saudi Arabia to investigate the association of immunomodulation and antiviral activity with black seed oil in hospitalized adult patients diagnosed with COVID-19 [39].

### 4.4. Peppermint

Peppermint (*M. piperita*) is among the oldest herbal remedies in the world. Dried peppermint has been gathered since at least 1000 BC, and its use has been reported in ancient Egypt, Greece, and traditional Chinese medicine [40]. The active compounds in peppermint are volatile oils, including menthol, menthone, menthofuran, isomenthone, limonene, cineole, menthyl acetate, isopulegol, pulegone, and carvone, in addition to flavonoid glycosides [41]. Menthol is used mainly to reduce inflammation, treat cold, cough, headache, digestive issues, and skin diseases, among many other uses [42]. A study conducted in Jazan, Saudi Arabia, investigated the antibacterial and antifungal activities of peppermint essential oils and found significant antibacterial and antifungal activity against Gram-negative and Gram-positive bacteria, yeast, and fungi, mainly as a result of the presence of the abundant phytochemicals menthol and menthone [43]. However, to the best of our knowledge, no study reported that peppermint had any antiviral activity against SARS-CoV-2. Regardless, this study found that 78% of nonhospitalized patients used peppermint, compared with only 22% of hospitalized patients, so that use of peppermint during infection with COVID-19 was associated with lower odds of hospitalization even after controlling for the effects of all other factors in the multivariable logistic regression (OR = 0.53; 95% CI: 0.31–0.90).

### 4.5. Honey

Beyond its religious and traditional associations, honey exhibits a wide range of pharmacological activities, including antioxidant, anti-inflammatory, antiviral, antineoplastic, antibacterial, antifungal, and wound healing properties [44]. Honey’s composition contains at least 181 substances, including fructose (38%) and glucose (31%). Enzymes, amino acids, proteins, flavonoids, and phenolic acids are also present. Riboflavin, niacin, folic acid, pantothenic acid, pyridoxine, and ascorbic acid are among the minor vitamins in honey [45]. A study was conducted in 2020 to review evidence of honey’s role as a potentially effective natural product against COVID-19. A previous study found that consuming honey might reduce the severity of COVID-19 infection, either directly, based on its potential antiviral effects against SARS-CoV-2, or indirectly, by boosting immune response [46]. Only about 43% of participants in the current study were using honey before contracting COVID-19, but 71% used it afterward. Although the proportion of patients hospitalized for COVID-19 treatment was significantly lower among honey users, this was not significant after controlling for the effect of all other factors in the multivariable logistic regression.

### 4.6. Ginger

Ginger (*Z. officinale*) is a medicinal plant that is widely used in traditional systems of medicine. The two principal pungent active constituents of ginger-based preparations are gingerol and shogaol. Ginger has antiemetic, antipyretic, analgesic, antiarthritic, antibacterial, antifungal, anti-inflammatory, and hepatoprotective effects. It also has a strong antioxidant effect that helps in the scavenging of free radicals [47,48,49,50]. In a study of 32 patients diagnosed with ARDS, 120 mg of ginger extract increased the tolerability of enteral feeding, decreased nosocomial pneumonia, and boosted ICU-free and ventilator-free days compared to patients who received placebo [51]. Another study that investigated the effect of ginger on inflammatory markers among patients treated for tuberculosis found the use of ginger to be associated with a significant reduction in the level of inflammatory markers, such as tumor necrosis factor-alpha (TNF-α) and ferritin [52]. Overall, these studies suggested that ginger extract could improve the health of people suffering from respiratory conditions, such as ARDS, pulmonary fibrosis, and pneumonia, or inflammatory conditions—progressions reported in patients with COVID-19. In the current study, participants’ use of ginger after infection showed a significant (*p* < 0.0001) 21% increase. Although the proportion of patients hospitalized for COVID-19 treatment was significantly lower among users of ginger than among nonusers (28% vs. 38%), this was not significant after controlling for the effect of all other factors in the study.

### 4.7. Turmeric

Turmeric (*C. longa*) is an integral part of some Asian cultures and cuisine. Curcumin, the predominant curcuminoid in turmeric, influences multiple signaling pathways and has been found to possess antioxidant, anti-inflammatory, antineoplastic, antiproliferative, and antimicrobial properties [53]. Turmeric is widely used as a home remedy for cough and sore throat and has been found to have an antiviral effect against different viruses. Moreover, curcumin is a natural ligand of peroxisome proliferator-activated receptor-gamma (PPAR-γ) and can reduce cytokine production, suggesting that it may play a role in protecting against lung injury associated with COVID-19 [54]. Although the proportion of patients hospitalized for COVID-19 treatment was significantly lower among users of turmeric than among nonusers (21% vs. 35%; *p* = 0.0012), this was not significant after controlling for the effects of all other variables in the study.

### 4.8. Garlic and Onion

Garlic (*Allium sativum*) and onion (*Allium cepa*) are commonly used as ingredients in Saudi cuisine. Onion, which has been used in traditional medicine for thousands of years to treat various conditions and infections, was found to destroy the avian influenza virus (H9N2) [55]. In 1919, onion was used as a home remedy against Spanish influenza in the United States [56]. However, preparation methods are crucial, as boiled or fried onions are fairly ineffective. Recent data suggest that onion may be a candidate to treat COVID-19 patients due to its anti-inflammatory, antithrombotic, and antiviral effects [56].

Garlic is well-known for its immunomodulatory, antimicrobial, antioxidant, anti-inflammatory, anticarcinogenic, antihypertensive, antithrombotic, antidiabetic, antimutagenic, and prebiotic activities. Secondary metabolites of garlic can be divided into sulfur-containing and sulfur-free active compounds. The main sulfur compounds are allicin and alliin, while the predominant sulfur-free active compounds include flavonoids and saponins [57]. Garlic’s ability to inhibit the SARS-CoV-2 was observed in silico concerning the formation of hydrogen bonds between amino acids in the binding site of the main structural protease of SARS-CoV-2 and garlic bioactive components that protease being responsible for viral replication [58]. Usually, patients who have COVID-19 have a decreased number of helper T cells, cytotoxic T cells, regulatory T cells, and natural killer (NK) cells, but increased leptin, TNF-α, interleukin-1 (IL-1), interleukin-2 (IL-2), and interferon-gamma (IFN-γ). Notably, consumption of garlic causes a significant increase in helper T cells, cytotoxic T cells, and NK cells, as well as a reduction in the levels of leptin, leptin receptor, TNF-α, IL-6, and proliferator-activated receptor gamma (PPAR-γ). Garlic could be another possible treatment for COVID-19 due to its ability to modulate cytokine secretion, immunoglobulin production, phagocytosis, and macrophage activation [59]. In the present study, a small increase in using garlic/onion was seen after infection (5%). The use of either was not associated with any impact on the proportion of patients hospitalized for COVID-19 treatment.

### 4.9. Costus, Anise, and Coffee Peels

Costus (*S. costus*) is widely known in prophetic medicine as well as in Ayurveda, Unani, and Siddha. It contains phytochemicals from diverse chemical classes, such as alkaloids, cardiac glycosides, coumarins, flavonoids, phenols, quinones, steroids, tannins, and terpenoids [60]. Costus has many pharmacological effects, including anti-cancer, anti-inflammatory, immunomodulatory, CNS depressant, and antimicrobial [61]. It is traditionally used to treat inflammation of the lungs, cough, cold, ulcer, and rheumatism [62]. Costus may have a potential role in COVID-19 treatment based on its use in treating fever, headache, cough, and bronchial asthma. In addition, the oleic acid in the costus acts as a bronchodilator, and the camphene, inulin, alpha-phellandrene, caryophyllene, and hexanoic acid act as expectorants. Moreover, costus has some complement-inhibitor substances and immunomodulatory effects on cytokine release, both of which can be helpful in treating diseases characterized by the presence of inflammatory markers [63]. The use of costus among the public during the pandemic has significantly increased. Moreover, the proportion of patients, who were hospitalized for COVID-19 treatment was significantly lower among users of costus than among nonusers (22% vs. 34%; *p* = 0.0095), although not at a significant level when adjusting for the effects of other variables.

Anise (*Pimpinella anisum*) is an aromatic plant, and the major components of its essential oil are anethole, eugenol, trans-anethole, methylchavicol, anisaldehyde, umbelliferon, estragole, coumarins, scopoletin, estriols, terpene hydrocarbons, polyenes, and polyacetylenes. Anise has been traditionally used as an analgesic, appetizer, sedative, expectorant, galactagogue, carminative, hepatoprotective, and disinfectant [64,65]. Additionally, it has been suggested that anise could have potential use in relieving early symptoms of COVID-19 because of its efficacy in relieving cough and fever [66]. In addition, its effect on asthma has been investigated in a clinical trial in which 50 asthmatic patients ingested an anise beverage (2 g of anise in 200 mL of water) twice a day for 40 days. After 21 days of treatment, all patients presented with a reduction in episodes of cough, dyspnea, and wheezing. They also experienced an improvement in breath-holding time and respiratory rate [66]. In the present study, a small increase in using anise was observed after infection (3%). However, using anise was not associated with any difference in the proportion of patients hospitalized for COVID-19 treatment.

Coffee (*C. arabica*) peels were among the avoided supplements identified in this study. Although only 74 patients (10%) consumed coffee peels before their COVID-19 infection, about 47% of these patients avoided their use during their infection. Moreover, the consumption of coffee peels was not associated with any impact on the hospitalization rate for COVID-19 treatment. Data supporting using coffee peels as an antiviral agent or in COVID-19 treatment or prevention are still lacking.

### 4.10. Limitations and Strength

Multiple limitations of this study should be emphasized. First, causality between the supplements used and the study outcome, hospitalization, was not established, as this was limited by the study design. Second, patients’ beliefs about the supplements in question were not assessed during the phone interview to keep the interview time within a reasonable range. Third, data on the number of supplements consumed by participants were not collected in this study. Thus, future studies may assess the smallest quantity of these supplements that could lead to some beneficial effects. Fourth, data on participants’ income and educational level were not obtained during data collection; thus, the analysis was not adjusted for the level of either one. However, the Saudi government, represented by the Saudi MoH, has declared free healthcare services for all COVID-19 patients. The limited number of studies examining the effect of dietary or herbal supplements on hospitalization for COVID-19 treatment in the Middle East and worldwide also limited our ability to compare our findings to others’. Accordingly, we structured our discussion to inform readers of why these specific supplements were evaluated. With these limitations in mind, the study reported data for a diverse sample of patients from different regions in Saudi Arabia, obtained through phone interviews, which can provide much the same experience as face-to-face interviews without requiring interviewers to travel or meet patients in different regions yet still produce nationally representative results. All this was achieved during the critical period of the COVID-19 pandemic, indicating that phone interviews can be a feasible option for future studies of public behaviors that involve distant geographic regions on a limited budget. As far as we know, this is the first study in Saudi Arabia to explore changes in the use of dietary or herbal supplements among individuals who contracted COVID-19 before and during their infection and to assess the association between the use of these supplements during infection with COVID 19 and subsequent hospitalization.

## 5. Conclusions

This study sheds light on changes in Saudi patients’ consumption of some dietary or herbal supplements before and during infection with SARS-CoV-2. We found a remarkable increase in the use of some dietary or herbal supplements. Further, we found that some patients who consumed some of these supplements were less likely to be hospitalized during their course of COVID-19 treatment. Accordingly, consumption of these supplements should be supervised by qualified healthcare providers to guarantee patient safety and to ensure that it is based on the best available evidence. In addition, the results of this study suggest some potential natural candidates that should be evaluated for further phytochemical and biological assessment in future studies of SARS-CoV-2.

## Figures and Tables

**Table 1:** Patient demographics.

Variables	Overall, Sample *N* = 738	Nonhospitalized *N* = 501	Hospitalized *N* = 237	*p*-Value *
**Age, mean (SD)**	36.5 (11.9)	33.6 (10.2)	42.4 (12.9)	**<0.0001**
**BMI, mean (SD)**	28.4 (7.1)	27.3 (7.1)	30.6 (6.8)	**<0.0001**
**Gender**	-	-	-	-
**Male**	418 (56.6)	286 (68.4)	132 (31.6)	0.7542
**Female**	320 (43.4)	215 (67.2)	105 (32.8)	-
**Nationality**	-	-	-	-
**Saudi**	559 (75.7)	409 (73.2)	150 (26.8)	**0.0012**
**Non-Saudi**	179 (24.3)	92 (51.4)	87 (48.6)	-
**Marital status**	-	-	-	-
**Single**	199 (27.0)	168 (84.4)	31 (15.6)	**0.0015**
**Married**	500 (67.8)	312 (62.4)	188 (37.6)	-
**Divorced**	26 (3.5)	18 (69.2)	8 (30.8)	-
**Widow/widower**	13 (1.7)	3 (23.1)	10 (76.9)	-

Data presented as frequency (%) unless otherwise indicated. * *p*-values were from t-test for continuous data (age and BMI) and chi-squared test for categorical data.

**Table 2:** Change in patients’ use of dietary or herbal supplements before and during infection with COVID-19.

Dietary or Herbal Supplements	Supplement Consumption before COVID-19 Infection		Supplement Consumption during COVID-19 Infection		Change in Use from before to during COVID-19 Infection	*p*-Value *
	Yes	No	Yes	No		
**Ginger**	267 (36.2)	471 (63.8)	425 (57.6)	313 (42.4)	158 (21.4%)	**<0.0001**
**Anise**	146 (19.8)	592 (80.2)	168 (22.8)	570 (77.2)	22 (3.0%)	**0.0482**
**Cumin**	82 (11.1)	656 (88.9)	77 (10.4)	661 (89.6)	−5 (−0.7%)	0.5529
**Chamomile**	54 (7.3)	684 (92.7)	51 (6.9)	687 (93.1)	−3 (−0.4%)	0.6911
**Peppermint**	260 (35.2)	478 (64.8)	161 (21.8)	577 (78.2)	−99 (−13.4%)	**<0.0001**
**Coffee peel**	74 (10.0)	664 (90.0)	50 (6.7)	688 (93.2)	−24 (−3.3%)	**0.0004**
**Lemon/orange**	368 (49.9)	370 (50.1)	591 (80.1)	147 (19.9)	223 (30.2%)	**<0.0001**
**Honey**	320 (43.4)	418 (56.6)	524 (71.0)	214 (29.0)	204 (27.6%)	**<0.0001**
**Black seed**	145 (19.6)	593 (80.4)	345 (46.8)	393 (53.2)	200 (27.1%)	**<0.0001**
**Costus**	17 (2.3)	721 (97.7)	115 (15.6)	623 (84.4)	98 (13.3%)	**<0.0001**
**Garlic/onion**	231 (31.3)	507 (68.7)	271 (36.7)	467 (63.3)	40 (5.4%)	**0.0078**
**Vitamin C**	360 (48.8)	378 (51.2)	505 (68.4)	233 (31.6)	145 (19.6%)	**<0.0001**
**Vitamin D**	255 (34.6)	483 (65.4)	259 (35.1)	479 (64.9)	4 (0.5%)	0.7335

Data presented as frequency (%); * *p*-value from McNemar’s test.

**Table 3:** Change in patients’ behavior during infection with COVID-19 for previous consumers or nonconsumers of dietary or herbal supplements before infection.

Natural Product or Supplement	When Had COVID-19	
	Avoided Use of Supplement ^‡^	Started Using Supplement
**Ginger**	43 (16.1)	201 (42.7)
**Anise**	51 (34.9)	73 (12.3)
**Cumin**	38 (46.3)	33 (5.0)
**Chamomile**	30 (55.6)	27 (4.0)
**Peppermint**	147 (56.5)	48 (10.0)
**Coffee peel**	35 (47.3)	11 (1.7)
**Lemon/orange**	40 (10.9)	263 (71.1)
**Honey**	43 (13.4)	247 (59.1)
**Black seed**	31 (21.4)	231 (39.0)
**Costus**	7 (41.2)	105 (14.6)
**Garlic/onion**	93 (40.3)	133 (26.2)
**Vitamin C**	16 (4.4)	161 (42.6)
**Vitamin D**	67 (26.3)	71 (14.7)

Data presented as frequency (%); ^‡^ patients who were using dietary or herbal supplement before they acquired COVID-19 but who avoided or did not use it afterward; patients who were not using dietary or herbal supplement before they acquired COVID-19 but who started using it afterward.

**Table 4:** Association between use of dietary or herbal supplements during infection with COVID-19 and hospitalization for COVID 19 treatment, before and after adjustment in multivariate logistic regression analysis.

Natural Product or Supplement	Patient Using Status during Infection	Nonhospitalized (*N* = 501)	Hospitalized (*N* = 237)	*p*-Value *	Odds Ratio (95%CI) ^†^
**Ginger**	Nonuser	195 (62.3)	118 (37.7)	**0.0053**	Reference
	User	306 (72.0)	119 (28.0)		1.06 (0.68–1.63)
**Anise**	Nonuser	379 (66.5)	191 (33.5)	0.1349	Reference
	User	122 (72.6)	46 (27.4)		1.11 (0.66–1.86)
**Cumin**	Nonuser	442 (66.9)	219 (33.1)	0.0827	Reference
	User	59 (76.6)	18 (23.4)		1.08 (0.49–2.36)
**Chamomile**	Nonuser	463 (67.4)	224 (32.6)	0.2937	Reference
	User	38 (74.5)	13 (25.5)		1.43 (0.62–3.32)
**Peppermint**	Nonuser	376 (65.2)	201 (34.8)	**0.0027**	Reference
	User	125 (77.6)	36 (22.4)		**0.53 (0.31–0.90)**
**Coffee peel**	Nonuser	466 (67.7)	222 (32.3)	0.7402	Reference
	User	35 (70.0)	15 (30.0)		1.39 (0.61–3.12)
**Lemon/orange**	Nonuser	75 (51.0)	72 (49.0)	**<0.0001**	Reference
	User	426 (72.0)	165 (28.0)		**0.54 (0.33–0.88)**
**Honey**	Nonuser	124 (57.9)	90 (42.1)	**0.0002**	Reference
	User	377 (72.0)	147 (28.0)		1.04 (0.64–1.70)
**Black seed**	Nonuser	241 (61.3)	152 (38.7)	**<0.0001**	Reference
	User	260 (75.4)	85 (24.6)		0.66 (0.42–1.05)
**Costus**	Nonuser	411 (66.0)	212 (34.0)	**0.0095**	Reference
	User	90 (78.3)	25 (21.7)		0.61 (0.33–1.12)
**Garlic/onion**	Nonuser	312 (66.8)	155 (33.2)	0.4108	Reference
	User	189 (69.7)	82 (30.3)		1.11 (0.70–1.69)
**Turmeric**	Nonuser	387 (65.2)	207 (34.8)	**0.0012**	Reference
	User	114 (79.2)	30 (20.8)		0.59 (0.33–1.06)
**Zinc**	Nonuser	355 (65.9)	184 (34.1)	0.0527	Reference
	User	146 (73.4)	53 (26.6)		0.68 (0.41–1.13)
**Vitamin C**	Nonuser	138 (59.2)	95 (40.8)	**0.0006**	Reference
	User	363 (71.9)	142 (28.1)		**0.51 (0.33–0.79)**
**Vitamin D**	Nonuser	336 (70.2)	143 (29.8)	0.0737	Reference
	User	165 (63.7)	94 (36.3)		**1.89 (1.20–2.98)**

Data presented as frequency (%); * *p*-value from chi-squared tests; ^†^ odds ratio from multivariate logistic regression with 95% confidence interval; the dependent variable in the logistic regression model was the need for hospitalization, and the results from the model were adjusted for the effects of age, gender, BMI, marital status, and the presence of any of the following comorbid conditions: diabetes, hypertension, dyslipidemia, pulmonary diseases (e.g., asthma, COPD), cardiovascular diseases, chronic kidney diseases, acquired immunodeficiency syndrome (AIDS), cancer, and depression or anxiety.

## Data Availability

The data are available upon reasonable request from the corresponding author.
